# MicroRNA Regulation of Endothelial Junction Proteins and Clinical Consequence

**DOI:** 10.1155/2016/5078627

**Published:** 2016-11-24

**Authors:** Yugang Zhuang, Hu Peng, Victoria Mastej, Weiguo Chen

**Affiliations:** ^1^Department of Emergency Medicine, Shanghai Tenth People's Hospital, Tongji University, Shanghai, China; ^2^Division of Pulmonary, Critical Care, Sleep and Allergy, Department of Medicine, University of Illinois College of Medicine, Chicago, IL, USA

## Abstract

Cellular junctions play a critical role in structural connection and signal communication between cells in various tissues. Although there are structural and functional varieties, cellular junctions include tight junctions, adherens junctions, focal adhesion junctions, and tissue specific junctions such as PECAM-1 junctions in endothelial cells (EC), desmosomes in epithelial cells, and hemidesmosomes in EC. Cellular junction dysfunction and deterioration are indicative of clinical diseases. MicroRNAs (miRNA) are ~20 nucleotide, noncoding RNAs that play an important role in posttranscriptional regulation for almost all genes. Unsurprisingly, miRNAs regulate junction protein gene expression and control junction structure integrity. In contrast, abnormal miRNA regulation of junction protein gene expression results in abnormal junction structure, causing related diseases. The major components of tight junctions include zonula occluden-1 (ZO-1), claudin-1, claudin-5, and occludin. The miRNA regulation of ZO-1 has been intensively investigated. ZO-1 and other tight junction proteins such as claudin-5 and occludin were positively regulated by miR-126, miR-107, and miR21 in different models. In contrast, ZO-1, claudin-5, and occludin were negatively regulated by miR-181a, miR-98, and miR150. Abnormal tight junction miRNA regulation accompanies cerebral middle artery ischemia, brain trauma, glioma metastasis, and so forth. The major components of adherens junctions include VE-cadherin, *β*-catenin, plakoglobin, P120, and vinculin. VE-cadherin and *β*-catenin were regulated by miR-9, miR-99b, miR-181a, and so forth. These regulations directly affect VE-cadherin-*β*-catenin complex stability and further affect embryo and tumor angiogenesis, vascular development, and so forth. miR-155 and miR-126 have been shown to regulate PECAM-1 and affect neutrophil rolling and EC junction integrity. In focal adhesion junctions, the major components are integrin *β*4, paxillin, and focal adhesion kinase (FAK). Integrin *β*4 has been regulated by miR-184, miR-205, and miR-9. Paxillin has been regulated by miR-137, miR-145, and miR-218 in different models. FAK has been regulated by miR-7, miR-138, and miR-135. Deregulation of miRNAs is caused by viral infections, tumorigenesis, and so forth. By regulation of posttranscription, miRNAs manipulate junction protein expression in all cellular processes and further determine cellular fate and development. Elucidation of these regulatory mechanisms will become a new alternative therapy for many diseases, such as cancers and inflammatory diseases.

## 1. Introduction

Cellular junctions are connective structures between cells existing in various tissues, such as epithelium, endothelium, and intestine. Although the cellular junction components and types may vary in different tissues, the majority of components in junctions are similar. Cellular junctions usually have tight junctions, adherens junctions, and focal adhesion junctions. In the endothelium, two identical PECAM-1s also form a junction and stabilize the major junctions. In addition to barrier function, some junction proteins, such as connexins, form a junction between cells which functions as an ion or water exchange channel (connexon). Integrins associate with focal adhesion kinase (FAK) and paxillin to form a focal adhesion complex, anchoring cells on the basal membrane. The focal adhesion complex also functions as a mechanical sensor which transduces extracellular signal into an intracellular downstream signaling pathway. Thus, these cellular junctions regulate cell function in their own ways.

MicroRNAs (miRNAs) are noncoding RNAs typically ~20 nucleotides in length and transcribed by RNA polymerase II from individual miRNA genes or coding gene introns. Most miRNAs are associated with RNA-induced silencing complex (RISC) and destabilize or inhibit target mRNA translation via binding to the 3′ untranslated terminal (3′UTR). miRNAs provide precise posttranscriptional control of target gene mRNA. Dysfunctional miRNA causes an abnormally high transcription level of the target mRNA and related clinical consequences. The large number and variable function of miRNA identified to date indicate that miRNAs are involved in a vast array of cellular processes, including development, growth, and tumorigenesis [1, 2]. However, the importance of miRNAs for cellular junction gene transcription has not been fully characterized.

Here, we review the important role of miRNAs in the processes of endothelial junction protein transcription and clinical relationships. It will help us to better understand the mechanism behind miRNA regulation of endothelial junctions and provide alternative therapies for the diseases caused by endothelial junction abnormality.

## 2. Tight Junction Proteins

### 2.1. Zonula Occludens-1 (ZO-1)/Occludin/Claudin-5

miRNA regulation of tight junctions has been systemically reviewed [3], and more miRNAs have been found involving tight junction expression. Moreover, the clinical consequence of miRNA regulation has not been reviewed, particularly endothelial tight junction regulation.

miRNA-107 abrogated the amyloid-beta (Abeta) protein-induced disruption of blood-brain barrier (BBB) and endothelial cell dysfunction. 3-UTR assay showed that endophilin-1 mRNA is the direct target of miR-107, which further affected endothelial junction by upregulation of ZO-1, occludin, and claudin-5 [4]. The rat permanent middle cerebral artery occlusion (MCAO) model showed that MCAO significantly increased BBB permeability in MCAO area. The expression of claudin-5 was decreased in MCA ischemic areas after upregulation of miR-150. Dual-luciferase assay confirmed that miR-150 directly regulated angiopoietin receptor Tie2 and then downregulated claudin-5 [5]. Deletion of miR-150 or knockout miR-150 caused irreversible increase in vascular permeability in mouse and cell model [6]. Interestingly, miR-21 increased in the brain following traumatic brain injury, promoting junction protein expression and exerting a protective effect on BBB by activating Ang-1/Tie2 axis [7].

In vitro and in vivo models showed that miR-98 negatively regulated tight junction proteins, such as ZO-1, by targeting hypoxia-inducible factor-1 (HIF-1) [8]. Diabetes (DM) rats had significant decrease in miR-126, claudin-5, occluding, and ZO-1 expression and worsened blood retinal barrier. Niaspan treatment reversed these deleterious effects and enhanced miR-126 level. This observation indicated that miR-126 plays an important role in the Niaspan-mediated antidiabetic effect and enhances endothelial barrier by targeting tight junction protein expression [9]. In glioma ECs, upregulation of miR-181a targeted Kruppel-like factor 6 (KLF6), downregulating ZO-1, occluding, and claudin-5, and increased permeability of the blood-tumor barrier (BTB) [10].

Similarly, by targeting runt-related transcriptional factor 1 (RUNX1), miR-18a downregulated mRNA of ZO-1, occludin, and claudin-5 in glioma vascular endothelial cells (GECs) and increased the permeability of BTB [11]. In metastatic breast cancer cells, miR-105 downregulated ZO-1 and increased endothelial barrier permeability, although the underlying mechanism was unclear [12]. By targeting HIV-1 Tat protein C (HIV-1 Tat C), miR-101 downregulated VE-cadherin, further downregulated claudin-5, and changed BMVECs permeability [13] (Table 1).

## 3. Adherens Junction Proteins

### 3.1. VE-Cadherin

The major components of adherens junctions include cadherins, *β*-catenin, plakoglobin, p120, and vinculin. These adherens proteins play an important role not only in cellular connection, but also in cellular signal exchange, contact-induced growth inhibition, and so forth [14]. Adherens junctions exist in all types of cellular connections and are relatively stable. As a major component of adherens junctions, E-cadherin (CDH1, existing in epithelium), VE-cadherin (CDH5, existing in vascular endothelium), and N-cadherin (CDH2, existing in neurons) have been extensively studied in past decades and miRNA regulation has been reported. miR-9 downregulated E-cadherin accompanying tumor metastasis or osteoblast differentiation [15, 16].

Early studies showed that miR-99b, miR-181a, and miR-181b potentiated the mRNA and protein expression of PECAM-1 and VE-cadherin accompanying differentiation of human embryonic stem cells to vascular endothelial cells [17]. miR-21 also regulated induced pluripotent stem cell (iPSC) differentiation into ECs by directly targeting VE-cadherin [18]. miR-125b inhibited VE-cadherin translation and in vitro tube formation by tumor ECs [19]. Overexpression of miR-142a-3p resulted in loss of vascular integrity, hemorrhage, and vascular remodeling during zebrafish embryonic development, while loss of function of miR-142a-3p caused abnormal vascular remodeling. MiR-142a-3p functions in part by directly repressing VE-cadherin [20]. VE-cadherin also played a role in HIV-associated neurological disorders. HIV-1 Tat protein C increased the expression of miR-101, which led to downregulation of VE-cadherin and further caused an adverse effect on blood-brain barrier integrity and permeability [13]. By directly targeting VE-cadherin, ectopic expression of miR-27a blocked capillary tube formation and angiogenesis [21]. MiR-302c and miR-26b-5p also showed similar function during hepatocarcinoma vascular tube formation [22, 23]. By regulation of signal pathways, such as TGF-*β* and TGF-*β*2 signal pathways, several miRNAs, such as miR-20a and miR-21, indirectly modulated adherens junction protein expression and endothelial-mesenchymal transition [18, 24] (Table 1).

### 3.2. *β*-Catenin

*β*-Catenin is not only a central molecule of many signaling pathways, such as the Wnt signaling pathway, but also a critical part of the VE-cadherin junction complex. By association with VE-cadherin, the *β*-catenin/VE-cadherin complex plays an important role in maintaining EC junction integrity. By far, of the majority of these studies focused on the role of miRNAs in tumorigenesis or EMT [15, 25]. For example, miR-1826 level was much lower in bladder cancer (BC) cell lines compared to normal bladder cell lines. Transfection of miR-1826 into BC cells inhibited BC cell viability, invasion, and migration by interfering with the VEGF-*β*-catenin-ERK signaling pathway [26]. Downregulation of miR-23b triggered glioma growth inhibition, induced apoptosis, and suppressed invasion by inhibition of *β*-catenin/Tcf4 and HIF-1*α*/VEGF signaling pathways [27]. Moreover, miRNA-184 downregulated *β*-catenin and deleteriously changed endothelial cell adherens junction structure and functions in HUVECs and HCE [28] (Table 1).

## 4. PECAM-1

PECAM-1 is specifically expressed in blood and endothelial cells. PECAM-1 on circulating platelets and leukocytes functions as an inhibitory receptor that limits cellular activation responses. However, PECAM-1 is highly expressed at endothelial cell intercellular junctions, where it functions as a mechanical sensor, regulator of leukocyte trafficking, and in the maintenance of endothelial junction integrity [29]. Inhibition of miR-155 improved neurological impairment of rats with cerebral infarction accompanying enhancement of PECAM-1 expression and EC junction integrity improvement [30]. Interestingly, polyphenolics inhibited high glucose-mediated inflammatory response and decreased PECAM-1 and ICAM-1 protein levels in human vascular endothelial cells (HUVECs). MicroRNA screens indicated that miR-126 may be modulated by polyphenolics as the underlying mechanism to inhibit expression of PECAM-1 and ICAM-1 [31]. A hyperoxia-induced bronchopulmonary dysplasia study resulted in lower levels of PECAM-1 mRNA and protein. MicroRNA screening indicated miRNAs may participate in the occurrence and development of bronchopulmonary dysplasia, including PECAM-1 expression. However, the underlying mechanism of miRNA-mediated PECAM-1 regulation and clinical consequence has not yet been determined [32] (Table 1).

## 5. Focal Adhesion Junction Proteins

### 5.1. Integrin *β*4

In EC focal adhesion junctions, the major components include integrin *β*4, paxillin, and focal adhesion kinase (FAK). Integrin *β*4 associates with integrin *α*6 to form a complex which anchors ECs on the basal membrane and functions as a mechanical sensor. Unlike other integrin *β* isoforms, integrin *β*4 has a unique long cytosolic domain which functions as a dock for tyrosine kinases, such as Src and Fny. Once integrin *β*4 receives stimulation from the basal membrane, its cytosolic domain recruits a tyrosine kinase and activates downstream signaling pathway. Single-nucleotide polymorphisms (SNPs) studies in breast cancer (BC) samples indicated that an A allele of the SNP rs 743554 in the integrin *β*4 gene has strong association with oestrogen receptor-negative tumors and it is believed to affect binding efficiency with microRNAs. Compared with wild type genotype carriers, those with the A allele have a poor survival significantly associated with aggressive tumor characteristics: high grade, lymph node metastasis, and high stage [33]. MicroRNAs miR-184 and miR-205 competitively bind to the complementary sequences within the 3′untranslated region (3′UTR) of integrin *β*4 mRNA. Mutated miR-184 failed to compete with miR-205 for the overlapping target sites on the 3′ UTRs of integrin *β*4 and resulted in familial severe keratoconus [34]. MiR-205 also regulated tumor cell basal membrane deposits of lamin-332 and its receptor integrin *β*4 [35]. Silico analysis showed that miR-9 also regulated integrin *β*4 mRNA but had no experimental results validating it [36] (Table 1).

### 5.2. Paxillin

Knockdown of paxillin or ectopic expression of miR-137 inhibited tumor growth and metastasis of colorectal cancer (CRC) cells in vivo. A dual-luciferase reporter gene assay validated paxillin as a direct target of miR-137 [37]. A similar inhibitory effect of miR-137 was also observed in a non-small cell lung cancer model [38]. MiR-145 also inhibited paxillin expression by binding to the paxillin mRNA 3′UTR. Higher expression of paxillin and lower expression of miR-145 were observed in colorectal cancer tissues than corresponding paracancerous tissue [39]. Among high-risk human papillomavirus (HPV) 16-infected oral cavity squamous cell carcinoma (OCSSC), HPV16/18 infection was negatively associated with miR-218 expression and positively associated with paxillin expression. This observation indicated there is an inverse relationship between miR-218 and paxillin in HPV-infected OCSCC [40]. More interestingly, microRNAs have been found to modulate paxillin expression by epigenetic mechanisms [41, 42]. Alternatively, paxillin also functions as a regulator of microRNA expression, such as miR-125b, to further regulate tumorigenesis [43] (Table 1).

### 5.3. Focal Adhesion Kinase (FAK)

Focal adhesion kinase (FAK) is the key component of the focal adhesion complex, which links focal adhesions to the cytosolic signaling pathway. By modification of FAK, such as phosphorylation or dephosphorylation of tyrosine or serine, FAK transduces extracellular stimuli into downstream signaling, further regulating the cytoskeleton and cellular junction. miR-7 has been shown directly targeting FAK and is negatively correlated with lymph node metastasis and tumor node metastasis staging in colon cancer (CC) [44, 45]. miR-543 level decreased in endometrial cancer cells and inversely correlated with mRNA levels of FAK and Twist homolog 1 (TWIST1) [46]. By binding with the 3′UTR of mRNA, miR-138 and miR-135 inhibited FAK protein expression in different cancer cell lines, such as HeLa cell, SW480, and A375 cell. Moreover, regulation of FAK expression by miR-135 and miR-138 also affected cancer cell invasion, drug sensitivity, and tumor growth in both in vitro and in vivo models [47]. Finally, by targeting an upstream kinase of FAK, some miRNAs indirectly regulate FAK phosphorylation status and further affect cell functions, such as junction integrity, cell growth, and migration [48] (Table 1).

## 6. Summary

Junction proteins are groups of structural proteins involved in various critical cellular processes, including cellular connection and signal communication, cell growth, and migration. So far, endothelial junction integrity has shown to be involved in many diseases, such as edema, inflammatory cell infiltration, and tumor cell invasion. Accordingly, comprehensive understanding of cell junction protein regulation is crucial for both junction function study and clinical therapy. Among all of the regulatory mechanisms of junction protein expression, miRNAs have emerged as particularly promising targets for their structural and functional characteristics. In this review, we systemically reviewed the miRNAs and their targeting proteins of tight junctions, adherens junctions, focal adhesion junctions, and PECAM-1 junctions. Although some miRNAs –junction protein regulatory relationships were observed in models other than endothelial junctions, they still provide insights into understanding the importance of these miRNAs (Table 1). Moreover, the phenotypes of these regulatory miRNAs on targeting junction proteins are also presented and certainly help us better understand the importance of these miRNAs in clinical regards.

## Figures and Tables

**Table 1:** The major components of EC junction and their targeting miRNAs.

Junction protein	Targeting miRNAs	Clinical consequence
ZO-1/occludin/claudin-5	miR-105, miR-181a, miR-18a, miR-21, miR-150, miR-101, miR-126	EC barrier disruption Tumorigenesis

VE-cadherin	miR-99b, miR-181a, miR-181b, miR-21, miR-125b, miR-142a-3p, miR-101, miR-27a, miR-302c, miR-26b-5p, miR-20a	EC barrier disruption Tumorigenesis
*β*-Catenin	miR-1826, miR-23b	Growth, apoptosis, invasion

PECAM-1	miR-126, miR-155	EC barrier disruption Tumorigenesis

Integrin *β*4	miR-184, miR-205, miR-9	Tumor metastasis
Paxillin	miR-137, miR-145, miR-218, miR-125b	Tumor metastasis Infection
FAK	miR-135, miR-138, miR-543	Tumorigenesis Tumor invasion
